# MePHD1 as a PHD-Finger Protein Negatively Regulates *ADP-Glucose Pyrophosphorylase Small Subunit1a* Gene in Cassava

**DOI:** 10.3390/ijms19092831

**Published:** 2018-09-19

**Authors:** Ping’an Ma, Xin Chen, Chen Liu, Zhiqiang Xia, Yu Song, Changying Zeng, Youzhi Li, Wenquan Wang

**Affiliations:** 1 Department of Tropical Crops genomics, The Institute of Tropical Bioscience and Biotechnology (ITBB), Chinese Academy of Tropical Agricultural Sciences (CATAS), Haikou 571101, China; pinganma@yahoo.com (P.M.); liuchenneo@163.com (C.L.); xiazhiqiang@itbb.org.cn (Z.X.); zengchangying@itbb.org.cn (C.Z.); 2 College of Biological Engineering, Henan University of Technology, Zhengzhou 450001, China; 3 Institute of Crop Germplasm Resources, Xinjiang Academy of Agricultural Sciences, Urumqi 830091, China; songyu150@163.com; 4 State Key Laboratory for Conservation and Utilization of Subtropical Agro-bioresources, Guangxi University, Nanning 530004, China; liyouzhigxu@163.com

**Keywords:** cassava, plant homeodomain transcription factor, ADP-glucose pyrophosphorylase, starch biosynthesis

## Abstract

ADP-glucose pyrophosphorylase (AGPase) is an important enzyme in the starch synthesis pathway. Its enzyme activity can determine the efficiency of starch biosynthesis. Cassava (*Manihot esculenta* Crantz) is the main staple crop worldwide and has a high starch content in its storage root. However, the inner regulatory mechanism of AGPase gene family is unclear. *MePHD1*; a plant homeodomain transcription factor; was isolated through a yeast one-hybrid screening using the promoter of *ADP-glucose pyrophosphorylase small subunit1a* (*MeAGPS1a*) as bait, and cassava storage root cDNA library as prey. This factor could bind to the *MeAGPS1a* promoter in vitro and in vivo, and its predicted binding region ranged from −400 bp to −201 bp, at the translation initiation site. The transcript level of *MePHD1* could be induced by five plant hormones, and a temperature of 42 °C. This was down-regulated during the maturation process of the storage root. MePHD1 protein could repress the promoter activity of *MeAGPS1a* gene by a dual-luciferase assay; which indicated that MePHD1 is a negative regulator for the transcript level of *MeAGPS1a* gene.

## 1. Introduction

Cassava (*Manihot esculenta* Crantz) is an important tuberous root crop in tropical and subtropical countries and is consumed by over 600 million people worldwide. Cassava starch is also a key resource in the food processing and bio-energy industries. ADP-glucose pyrophosphorylase (AGPase) is the enzyme that starts the starch biosynthesis, and its enzyme activity can determine the efficiency of starch synthesis [1]. AGPase is a heterotetramer (α_2_β_2_) composed of two small/catalytic subunits and two large/modulatory subunits. *MeAGPS1a* is the most important small subunit in the cassava AGPase gene family and can be modulated by MeSAUR1 [2].

Plant homeodomain (PHD) transcription factors are important categories in eukaryotes because they are crucial for the regulation of gene transcription and chromatin state [3]. The PHD protein has one or several PHD-finger motifs, and a PHD-finger motif is composed of approximately sixty amino acid residues with conserved Cys4-His-Cys3, for zinc binding [4]. The PHD finger gene family has various members in different plants, e.g., seventy members in *Arabidopsis* [5], one hundred and six putative members in carrot [6], sixty-seven members in corn [7], seventy-three members in poplar [8], and sixteen members in soybean [9]. Several PHD members respond to high salinity, drought, and cold stresses. For instance, transgenic *Arabidopsis* plants, which overexpress the gene *GmPHD2*, show higher salt tolerance when comparing with its wild type [10]. GmPHD5 also acts as an important regulator for crosstalk between histone H3K4 di-methylation and H3K14 acetylation, in response to salinity stress in soybean [11]. Furthermore, MMD1, as a PHD transcription factor, is required for male meiosis, and its mutation can trigger cell death in *Arabidopsis* male meiocytes [12]. *MALE STERILITY1* in barley and *MALE STERILITY7* in corn both encode PHD-finger transcription factor [13,14].

In the present study, a PHD transcription factor gene, called *MePHD1*, was isolated from *Manihot*, through the yeast one-hybrid assay [2]. The MePHD1 protein could bind to the *MeAGPS1a* promoter, in vitro and in vivo. Moreover, MePHD1 protein represses the promoter activity of *MeAGPS1a*, which indicates that MePHD1 is a negative regulator of *MeAGPS1a* and could affect starch biosynthesis in cassava.

## 2. Results

### 2.1. Isolation and Molecular Characterization of MePHD1

**Figure 1:**
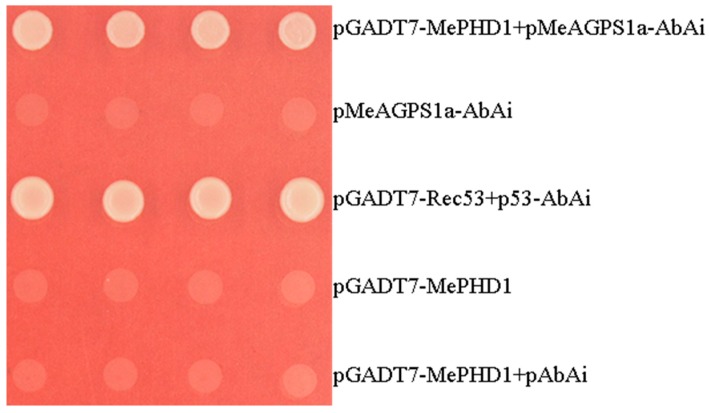
Activation of *MeAGPS1a* promoter, in yeast, by MePHD1. pGADT7-MePHD1 + pMeAGPS1a-AbAi is the experiment, pMeAGPS1a-AbAi is the empty vector control, pGADT7-Rec53 + p53-AbAi is the positive control, pGADT7-MePHD1 and pGADT7-MePHD1 + pAbAi are the negative controls. Yeast cells were grown in SD/-Leu selective medium, containing 300 ng/mL Aureobasidin A (AbA), for 3 days, at 30 °C.

**Figure 2:**
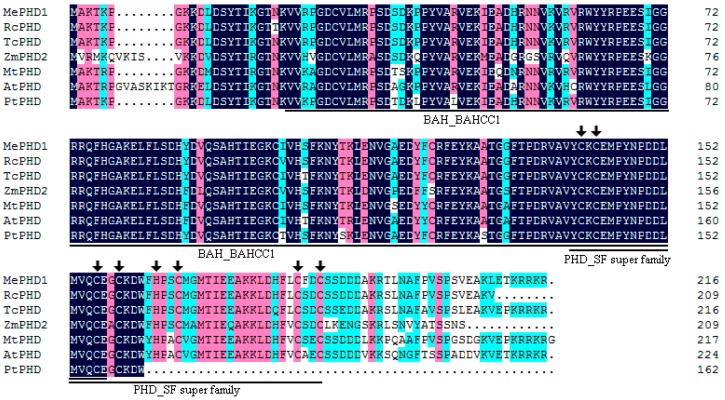
The PHD protein has one or several PHD-finger motifs, and a PHD-finger motif is composed of unique Cys4-His-Cys3 pattern zinc-containing domain. Amino acid sequences comparison of MePHD1 with related PHD proteins. Sequences were aligned by using DNAMAN6.0. Identical residues are highlighted in dark blue, and biochemically conserved substitutions are highlighted in two gradual darker colors (pink and cyan) according to identity. The conserved Cys4-His-Cys3 is indicated by the black arrow. Aligned sequences include MePHD1 (Manes.12G071000), RcPHD (B9SH49), TcPHD (XP_017969599), ZmPHD2 (NP_001131656), MtPHD (XP_013464613), AtPHD (NP_001031695), and PtPHD (G1FLG6).

AGPase is the first enzyme in starch biosynthesis in plants, and is composed of two small subunits and two large subunits. Only the cassava ADP-glucose pyrophosphorylase small subunit1a protein can interact with the large subunits, in the AGPase family. *MeAGPS1a* gene (Manes.12G067900) shows relatively high transcript level in the storage root of cassava. In the previous report, we used yeast one-hybrid assay to screen candidate transcription factors that could bind to the *MeAGPS1a* promoter. One of the transcription factors, MeSAUR1, could up-regulate the expression level of *MeAGPS1a* gene [2]. In the present study, MePHD1, a PHD-finger transcription factor (Manes.12G071000), which could also bind to *MeAGPS1a* promoter (Figure 1), was introduced. Its full-length transcript was 1482 bp long, including a 651 bp coding sequence, 265 bp 5′UTR and 566 bp 3′UTR, and it coded for a protein of 216 amino acid residues with a molecular mass of 24.8 KD. MePHD1 protein contained a BAH_BAHCC1 (Bromo Adjacent Homology-BAH domain and coiled-coil containing 1) conserved domain, and a PHD_SF (plant homeodomain super family) domain (Figure 2). Cassava PHD-finger (BAH) transcription factor family had 8 members, which were distributed on Chromosomes 4 and 12. *MePHD1* was located on Chromosome 12 (Figure S1 and Table S1). Furthermore, six types of *cis*-element were present in the 2000 bp promoter of *MePHD1* (e.g., two heat stress elements (HSEs), six auxin responsive elements (AREs), and five GCC boxes (Table S2)), which implied that the treatments of plant hormone and heat could affect the transcript level of *MePHD1*.

### 2.2. Transcript Profiles of MePHD1

**Figure 3:**
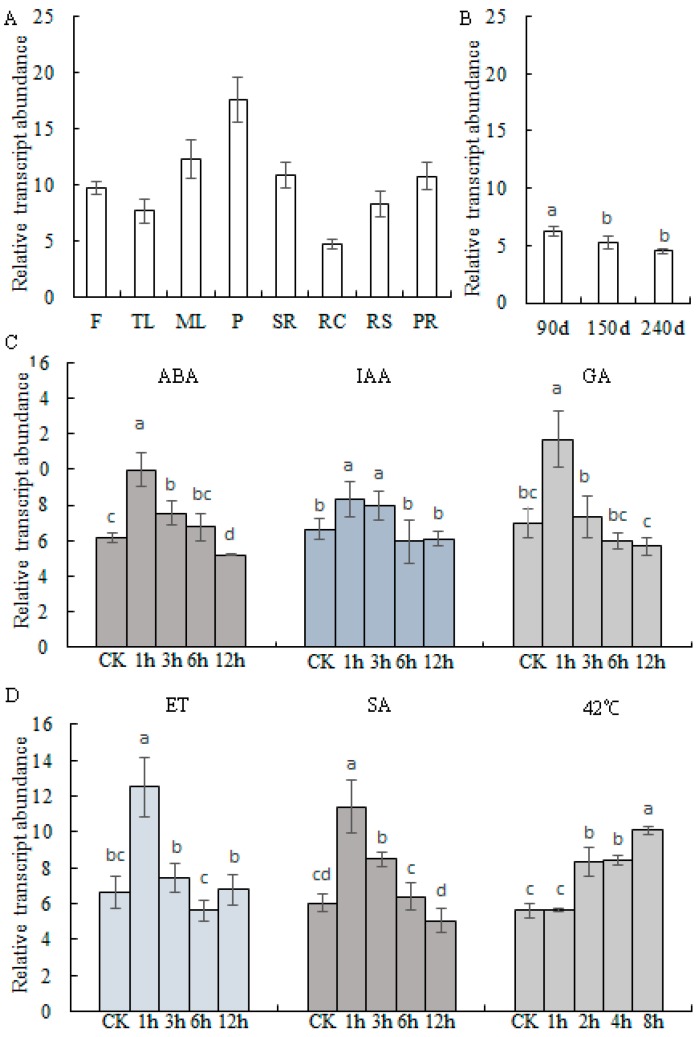
Transcription profiles of *MePHD1*. (**A**) Expression profile of *MePHD1* in different organs of cassava plant. F: Flower; TL: Tip leaf; ML: Mature leaf; P: Petiole; SR: Stem rind; RC: Root cortex; RS: Root stele; PR: Primal root. (**B**) Expression profile of *MePHD1* in three growth stages of storage root. (**C**,**D**) Transcription profiles of *MePHD1* respond to abscisic acid (ABA), indole-3-acetic acid (IAA), gibberellin (GA), Ethylene (ET), Salicylic acid (SA), and heat (42 °C). CK: The one-month-old tissue cultured plantlets of KU50. The y axis is the scale of the relative expression level. Error bars represent the SD of four technical replicates, the significant difference was assessed by ANOVA, a–c means the significant difference at *p* < 0.05 level.

qPCR assay was performed to investigate the transcript profiles of *MePHD1* in different cassava organs. The results suggested that *MePHD1* was highly expressed in the petiole, relatively less expressed in the root cortex, and moderately expressed in other organs (e.g., mature leaf, stem rid, and root stele (Figure 3A)), and down-regulated during the mature process of the storage root (Figure 3B). According to the distribution of *cis*-elements in the *MePHD1* promoter, cassava tissue cultured seedlings were treated by heat and plant hormones, including ABA, IAA, GA, SA, and ET. The heat treatment at 42 °C, up-regulated the transcript level of *MePHD1*, starting from 2 h after treatment, and continued up to 8 h. Five plant hormones also up-regulated the expression level of *MePHD1*, reached the highest value at 1 h post-treatment, and then fell down, starting from 3 h to 12 h (Figure 3C,D).

### 2.3. Subcellular Location of MePHD1 Protein

**Figure 4:**
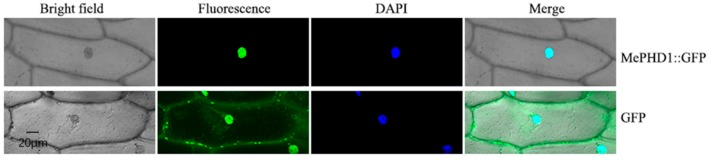
Nuclear localization of MePHD1. Top row/bottom row: the corresponding bright field, fluorescence, merged fluorescence image, and DAPI image of MePHD1-GFP/GFP control. Bar = 20 μm.

To ensure the location of the MePHD1 protein, in the cell, the green fluorescent protein (GFP) was fused with the GFP gene, in the frame, to the 5′ end of the MePHD1 gene. This was transiently transformed into the onion epidermal cells by the *Agrobacterium* line LBA4404, harboring a plasmid with MePHD1-GFP, and then observed under a confocal microscope. The MePHD1-GFP protein was located in the nucleus of the onion epidermal cell, whereas the control GFP was dispersed in the entire cell (Figure 4). The result precisely demonstrated that MePHD1 was a nucleus-localized protein.

### 2.4. MePHD1 Binds to MeAGPS1a Promoter In Vitro

**Figure 5:**
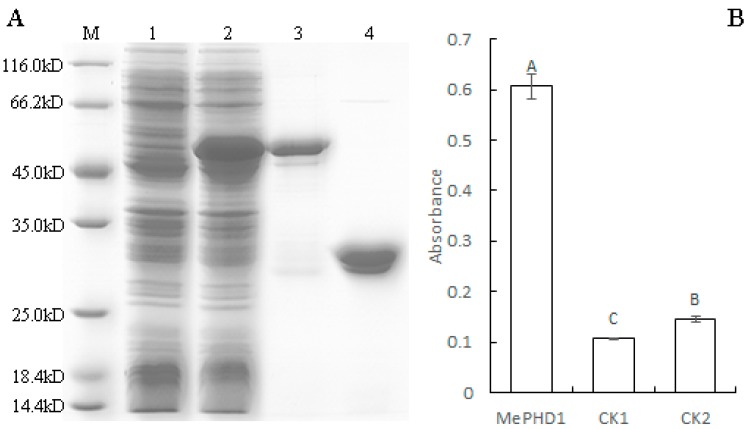
(**A**) MePHD1 binding to the promoter of *MeAGPS1a*, as analyzed DPI-ELISA. Over-expression of MePHD1 in *Escherichia coli*. M: Molecular markers; 1 represents the *E. coli* cells harboring pCold Pros2-MePHD1 not induced; 2 shows the *E. coli* cells harboring pCold Pros2-MePHD1, after 20 h of induction; 3 shows the purified MePHD1 fusion protein by the *E. coli* cells harboring pCold Pros2-MePHD1, after 20 h of induction; 4 shows the purified ProS2 tag protein, by the *E. coli* cells harboring pCold Pros2, after 20 h of induction. (**B**) MePHD1 interacts with the *MeAGPS1a* promoter by DPI-ELISA assay. MePHD1: Double-strand biotinylated *MeAGPS1a* promoter DNA probe + purified MePHD1 fusion protein; CK1: Double-strand biotinylated *MeAGPS1a* promoter DNA probe + tagged protein ProS2; CK2: Purified water + purified MePHD1 fusion protein. Error bars represent the SD of four technical replicates, the significant difference was assessed by ANOVA, A–C means the significant difference at *p* < 0.01 level.

To investigate whether the MePHD1 protein interacts with the promoter of *MeAGPS1a*, in vitro, the purified recombinant ProS2-MePHD1 protein was extracted from *Escherichia coli* harboring vector pCold Pros2-MePHD1, and its molecular mass matched with the theoretical value when the 23 KD tagged protein was added (Figure 5A). Then, the DNA-protein-interaction enzyme-linked immunosorbent assay (DPI-ELISA) was performed with the recombinant protein and the biotinylated *MeAGPS1a* promoter DNA probe. The recombinant protein bound to the *MeAGPS1a* promoter and the absorbance of MePHD1 + *MeAGPS1a* promoter was over 4-fold, as compared with those of the two controls (Figure 5B). The results indicated that MePHD1 could bind to the promoter of *MeAGPS1a*, in vitro.

### 2.5. MePHD1 Binds to the −400 bp to −201 bp Region of MeAGPS1a Promoter

**Figure 6:**
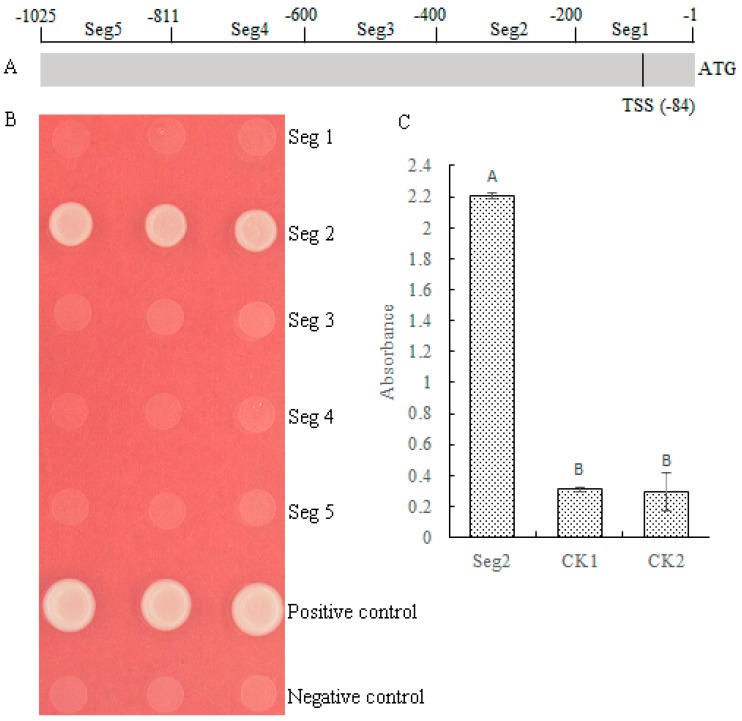
(**A**) MePHD1 binding to the region of −400 to −201 in *MeAGPS1a* promoter. Distribution of five segments in the *MeAGPS1a* promoter. TSS: Transcription start site. ATG: Initiation codon. (**B**) Activation of *MeAGPS1a*, promoter in yeast, by MePHD1. Yeast cells carried pGADT7-MePHD1+p(Seg1 to Seg5)-AbAi, positive control (pGADT7-Rec53+p53-AbAi), and negative control (pGADT7-MePHD1 + pAbAi), were grown in SD/-Leu selective medium with 50 ng/ml AbA, for 3 days at 30 °C; the three dots in each line mean three replications. (**C**) MePHD1 interacts with the Seg2 by DPI-ELISA assay. Seg2: Double-strand biotinylated Seg2 DNA probe + purified MePHD1 fusion protein; CK1: Double-strand biotinylated Seg2 DNA probe + tagged protein ProS2; CK2: Purified water + purified MePHD1 fusion protein. Error bars represent the SD of four technical replicates, the significant difference was assessed by ANOVA, A,B means the significant difference at *p* < 0.01 level.

To determine the exact binding region of MePHD1 protein, the *MeAGPS1a* promoter was cleaved into five segments. Each segment was 200 bp long and was called Seg1 to Seg5 (Figure 6A). The Y1H Gold yeast strain was transformed by pAbAi vectors carrying the segments of Seg1 to Seg5, respectively. Then, the pGADT7-AD-MePHD1 was transformed into pSeg1-AbAi-pSeg5-AbAi yeast cells, which was similar to the method used by Ma et al. [2]. Only the pGADT7-AD-MePHD1 + pSeg2-AbAi yeast cells could grow well in the SD/-Leu selective medium containing 50 ng/mL AbA (Figure 6B). Afterward, DPI-ELISA assay was again performed using the Seg2 probe and the recombinant MePHD1 protein. The results implied that MePHD1 could exactly bind to the −400 bp to −201 bp region of *MeAGPS1a* promoter (Figure 6C).

### 2.6. Repression of MeAGPS1a Promoter by MePHD1 Protein

**Figure 7:**
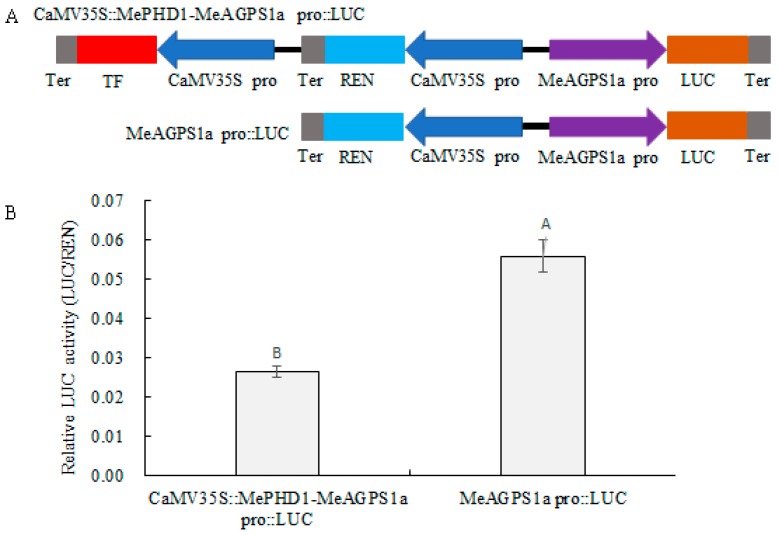
Repression of *MeAGPS1a* promoter, in the transient expression system, by MePHD1. (**A**) Schematic diagrams of the two transient expression vectors. (**B**) Relative LUC activity of vector pCaMV35S::MePHD1-MeAGPS1a Pro::LUC and pMeAGPS1a Pro::LUC, in tobacco leaves. Error bars represent the SD of nine technical replicates, the significant difference was assessed by ANOVA, A,B means the significant difference at *p* < 0.01 level. TF: Transcription factor; REN: Renilla luciferase; LUC: Luciferase; pro: *MeAGPS1a* promoter; Ter: Terminator.

A dual-LUC reporter analysis was adopted to investigate the regulation of the transcription of the *MeAGPS1a* gene by MePHD1. First, a binary expression vector was constructed, which had two expression cassettes of 35S::MePHD1 and pMeAGPS1a::LUC, and another vector was used, which had only one expression cassette of pMeAGPS1a::LUC, along with 35S::REN acting as the control (Figure 7). Second, tobacco leaf epidermal cells were infiltrated by *Agrobacterium tumefaciens* LBA4404, harboring the two vectors described above, respectively. After checking the values of relative luciferase activity (LUC/REN) in the total protein of tobacco leaves, LUC/REN value of 35S::MePHD1 + pMeAGPS1a::LUC was less than that of pMeAGPS1a::LUC, thereby, suggesting that MePHD1 could repress the transcript level of the *MeAGPS1a* gene in the cassava plant.

## 3. Discussion

This study focused on the MePHD1, a PHD-finger protein, which was screened out by a yeast one-hybrid assay. MePHD1 is a nucleus-localized protein that could interact with the *MeAGPS1a* promoter, in vitro and in vivo, and could repress the promoter activity of *MeAGPS1a* gene. The transcript level of *MePHD1* could be induced by the application of exogenous plant hormones and heat stress, and the relevant *cis*-elements that existed in the *MePHD1* promoter.

Several reports on the PHD-finger proteins concentrated on the abiotic stress resistance and the plant development, whereas, only a few reports paid attention to its function in regulating starch biosynthesis. The transcript level of *AL5*, an alfin-like PHD-finger gene, responded to the abiotic stresses and the abscisic acid (ABA) treatment. *AL5*-over-expression lines showed higher tolerance to salt, drought, and freezing stresses than the wild type. Meanwhile, *al5* mutants showed reduced stress tolerance, which indicated that AL5 inhibited multiple signaling pathways to confer stress tolerance [15]. *Arabidopsis EBS* gene, like *MePHD1*, is also an EBS-like protein, which participates in the developmental processes as a negative regulator, especially in regulating the flowering time by repressing the expression of *FT*, a key gene in the integration of floral promotion pathways [16,17], and even in the regulation of seed dormancy [18]. Furthermore, the expression level of *MePHD1* could be induced by five kinds of plant hormones, especially for heat stress, which could up-regulate the transcript level of *MePHD1* steadily. This finding suggested that MePHD1 might be a hot point in multiple signaling pathways in cassava, except for its regulation of the *MeAGPS1a* gene.

DPI-ELISA is a new technique to detect the interaction between DNA and protein, through immunoreaction. Compared with the traditional electrophoretic mobility shift assay, DPI-ELISA has more advantages because it does not rely on radioactive detection. In addition, DPI-ELISA displays a 10-fold increase in sensitivity, is cost-effective, requires less time-consumption, and provides qualitative and quantitative readouts [19]. In cassava, Ma et al. [2] confirmed the interaction between MeSAUR1 protein and *MeAGPS1a* promoter with DPI-ELISA, according to other relative experiment results. Therefore, DPI-ELISA might be a new alternative to assay the interaction between DNA and protein, especially for non-model plants.

## 4. Materials and Methods

### 4.1. Plant Materials

*Manihot esculenta* Crantz cv. KU50 was planted in the experimental field of the Institute of Tropical Biosciences and Biotechnology, Chinese Academy of Tropical Agricultural Sciences (Chengmai County, Hainan Province, China). The tip and mature leaves, flowers, petioles, stem rinds, root steles, root cortices, tuber roots, and primal roots were collected at 180 days after planting (DAP). The three growth stages of storage root were collected at 90, 150 and 240 DAP. The one-month-old tissue cultured plantlets of KU50 was set under a culture condition of 25 °C, for 16/8 h in light/dark photoperiod, and treated with 100 μM abscisic acid (ABA), 10 mM Ethephon (2-chloroethyl phosphoric acid, ET), 100 μM Gibberellin A3 (GA3), 10 μM 3-Indole acetic acid (IAA, Sigma, San Francisco, CA, USA), and 5 mM salicylic acid (SA). The leaves were harvested at 1, 3, 6, and 12 h after treatments. Furthermore, the plantlets were incubated at 42 °C, and the leaves were collected after 1, 2, 4, and 8 h. Each sample contained nine plantlets. The untreated plantlets were collected as control. All materials were immediately stored at −80 °C, after liquid-nitrogen-freezing.

### 4.2. Yeast One-Hybrid

Yeast one-hybrid assay was performed according to the method described by Li et al. [20]. The bait vector pAbAi-MeAGPS1a was constructed for yeast one-hybrid assay, and the cassava storage root total RNA was isolated. Afterward, double-stranded cDNA was synthesized as described in our previous study [2]. The double-stranded cDNAs were linked with *Sma* I linearized prey vector pGADT7-Rec, transformed into the yeast positive strain Y1HGold/pAbAi-MeAGPS1a (Clontech, Mountain View, CA, USA), and then cultivated on SD/-Leu medium with 300 ng/mL Aureobasidin A (AbA), at 30 °C, for 3 days. The positive colonies were identified by plasmid polymerase chain reaction and sequence analysis.

To confirm the interaction between MePHD1 and *MeAGPS1a* promoter, the *MePHD1* coding sequence (CDS) was amplified with P1 primer pair (forward: 5′-CATATGGCCAAAACCAAACCAG-3′ and reverse: 5′-GTCGACTCTCTTCCTTCGCT-3′). The *MePHD1* CDS was ligated into a pGADT7 vector, through *Nde* I and *Sal* I, and named pGADT7-MePHD1. pGADT7-MePHD1 and pAbAi-MeAGPS1a were co-transformed into the Y1HGold yeast strains. pGADT7-Rec53 + p53-AbAi, pAbAi-MeAGPS1a, pGADT7-MePHD1, and pGADT7-MePHD1 + pAbAi were used as controls. The transformed cells were grown on SD/-Leu selective medium with 300 ng/mL AbA, at 30 °C for 3 days.

### 4.3. Chromosomal Location and Bioinformatic Analyses of the MePHD Gene Family

Cassava PHD genes and location information were derived from Phytozome 12 database (Available online: https://phytozome.jgi.doe.gov). The chromosomal locations of cassava PHD genes were mapped by MapInspect software (Available online: http://mapinspect.software.informer.com). The PHD amino acid sequences of other species were obtained by the BLAST program of NCBI (Available online: https://www.ncbi.nlm.nih.gov/) and UniPort (Available online: http://www.uniprot.org/) databases, using MePHD1 as a query sequence. Amino acid comparison was performed using DNAMAN software (Lynnon Biosoft, San Ramon, CA, USA).

### 4.4. Quantitative Polymerase Chain Reaction (qPCR)

Total RNA was extracted using plant RNeasy extraction kit (Tiangen, Beijing, China). RevertAidTM First-Strand cDNA Synthesis Kit (Fermentas, Ontario, Canada) was used to synthesize the First-Strand cDNA. QPCR was performed according to the method of Hu et al. [21], using Stratagene Mx3000P Real-Time PCR and SYBR^®^ Premix Ex Taq™ (TaKaRa, Tokyo, Japan). The qPCR of *MePHD1* was conducted with the primers Rt*PHD1*F (5′-GGTACTATCGGCCAGAGGAG-3′) and Rt*PHD1*R (5′-CAGAAGTAGTCCTCAGCTCCA-3′). A housekeeping gene β-actin was used as a standard control which was amplified by primers *Actin*F (5′-CAAGGGCAACATATGCAAGC-3′) and *Actin*R (5′-CCTTCGTCTGGACCTTGCTG-3′). The PCR amplification conditions were as follows: 90 s at 95 °C for denaturation, and 40 cycles of 10 s at 95 °C, 15 s at 55 °C, and 30 s at 72 °C, for amplification. The relative gene expression data were calculated based on the 2^−ΔΔCt^ method [22]. Four independent biological replicates of each sample were implemented, and significant difference analysis was tested with IBM SPSS Statistics 23 software (IBM, Armonk, NY, USA).

### 4.5. Nuclear Localization Analysis

The full-length coding sequence of *MePHD1* was amplified with forward primer 5′-GGATCCATGGCCAAAACCAA-3′ and reverse primer 5′-GTCGACTCTCTTCCTTCGCT-3′, by using the single-strand cDNAs. This reverse transcript from KU50 total RNA, using it as a template, and contained *Bam* HI and *Sal* I sites (underlined). The coding sequence of MePHD1 was introduced into the pCAMBIA1302 vector through the *Bam* HI/*Sal* I sites and generated CaMV35S::MePHD1-GFP. Then, the CaMV35S::MePHD1-GFP and pCAMBIA1302 were transformed into *Agrobacterium tumefaciens* and the onion inner epidermal cells were infected. The transformed epidermal cells were cultured on an MS medium, at 25 °C, for 36 h without light, and then observed using confocal microscopy.

### 4.6. MePHD1 Protein Expression and Purification

To get a large amount of MePHD1 protein, the full-length coding sequence of MePHD1 was amplified with P1 primer pairs and then fused to the *Nde* I/*Sal* I sites in the pCold Pros2 vector (TaKaRa, Tokyo, Japan), which generated the pCold Pros2-MePHD1. pCold Pros2-MePHD1 was introduced into *Escherichia coli* strain BL21 (DE3) competent cells for protein expression. The transformed strains were cultured in LB medium supplied with 100 mg/L Ampicillin, at 37 °C, and 250 rpm until the OD600 of the culture reached 0.4–0.8, and the bacterial cell fluid temperature cooled down to 15 °C. The cultures were incubated at 15 °C and 120 rpm, for 24 h, after adding 1.0 mM isopropyl β-d-1-thiogalactopyranoside. The soluble proteins of the cultured cells were extracted and purified by BugBuster^®^ Protein Extraction Reagent (Novagen, Darmstadt, Germany) and Ni-charged MagBeads (GenScript, Jiangsu, China), respectively. pCold Pros2 was expressed and purified as control. The purified protein was verified by 12% sodium dodecyl sulfate polyacrylamide gel electrophoresis. When performing Western blotting, Tag Anti-ProS2 (TaKaRa, Tokyo, Japan) and Goat Anti-Mouse IgG/HRP (Boster, Wuhan, China) were used as primary and secondary antibodies, respectively.

### 4.7. DNA-Protein-Interaction Enzyme-Linked Immunosorbent Assay (DPI-ELISA)

DPI-ELISA was carried out according to the method described by Brand et al. [19]. The biotinylated *MeAGPS1a* promoter probe was obtained by PCR, with Biotin-*MeAGPS1a* primer pairs and KU50 genomic DNA as template (forward: 5′-Biotin-CAGCTGCCCCTACCGTTAA-3′ and reverse: 5′-Biotin-TAGCAAGTTCAGATTTGGAAAAAACC-3′). The ELISA micro-well plates used Pierce^®^ Streptavidin High Capacity Coated Plates (Thermo Fisher Scientific, Rockford, IL, USA). Tag Anti-ProS2 (TaKaRa, Tokyo, Japan) and Goat Anti-Mouse IgG/HRP (Boster, Wuhan, China) were used as the primary and secondary antibodies in the DPI-ELISA.

### 4.8. Dual-Luciferase (Dual-LUC) Assay

The Dual-LUC assay was performed by following the method of Hellens et al. [23]. Both LUC and REN came from pGreenII0800-LUC, in this research. The MeAGPS1a promoter was obtained with Pro-MeAGPS1a primers (forward: 5′-GAATTCCAGCTGCCCCTACC-3′ and reverse: 5′-ACTAGTTAGCAAGTTCAGATTTGGAAAA-3′), by PCR. The PCR production was ligated with LUC through *EcoR* I/*Spe* I (underlined), which generated MeAGPS1a pro::LUC. The MePHD1 CDS amplified in the nuclear localization section was fused with CaMV35 promoter and ligated with MeAGPS1a pro::LUC to generate CaMV35S::MePHD1-MeAGPS1a pro::LUC. Two constructed vectors were introduced into *A. tumefaciens* strain BLA4404. The transformed strains were cultured in LB (50 mg/L Kanamycin + 50 mg/L Rifampicin) medium at 37 °C and 250 rpm, until the OD600 of the culture reached 0.6–1.0. Subsequently, the culture was infiltrated into the abaxial side of the tobacco leaves. Three days after infiltrating, the total protein was extracted from the infected area of the tobacco leaves. The fluorescent values of LUC and REN were measured according to the manual of Dual-luciferase Reporter Assay System (Promega, Madison, WI, USA). Each sample had 16 replicates, and about 8–10 repetitions that were close to the median value, were retained for analysis.

## Supplementary Materials

Supplementary materials can be found at http://www.mdpi.com/1422-0067/19/9/2831/s1.
