# Supplementary material for: MePHD1 as a PHD-Finger Protein Negatively Regulates ADP-Glucose Pyrophosphorylase Small Subunit1a Gene in Cassava
Source: Int J Mol Sci. 2018 Sep 19;19(9):2831. doi: 10.3390/ijms19092831 (PMC6164933; doi:10.3390/ijms19092831)
Supplement: Supplementary file 1 [file ijms-19-02831-s001.zip › ijms-345535 legends.pdf]

MePHD1 as a PHD-Finger Protein Negatively Regulates *ADP-Glucose Pyrophosphorylase Small Subunit1a* Gene in Cassava

Figure S1. Distribution of *MePHD* members in the cassava genome. The 125 cassava PHD genes were mapped to the 18 chromosomes, except Manes.S022700.1 located on unassembled scaffolds and their gene IDs are listed in Supplementary Table S1. Chromosome numbers are indicated at the top of each bar. The left scale represents the Megabase (Mb). The gene IDs on the left in each Chromosome. Boldface represents eight PHD-finger (BAH) transcription factors and red font represents *MePHD1.*

Table S1. Listed members and functional annotation of 126 MePHD proteins.

Table S2. *Cis*-elements distribution in the promoter of MePHD1 related to four plant hormones.
