# Supplementary material for: MePHD1 as a PHD-Finger Protein Negatively Regulates ADP-Glucose Pyrophosphorylase Small Subunit1a Gene in Cassava
Source: Int J Mol Sci. 2018 Sep 19;19(9):2831. doi: 10.3390/ijms19092831 (PMC6164933; doi:10.3390/ijms19092831)
Supplement: Supplementary file 1 [file ijms-19-02831-s001.zip › Table S2.pdf]

Table S2. *Cis*-element distribution in the promoter of *MePHD1* related to heat and plant hormones.

Cis-element	Position/Strand	Sequence	Expected function
HSE	−1232(+),−132(+)	AAAAAATTTT/A AAAAAATTC	Heat stress-responsive element
ABRE	−409(+)	CACGTG	ABA-responsive element
ARE	−956(+),−854(+),−691(+),−417(+),−405(+),−11(+)	TGTCNN	Auxine-responsive element
ERE/GCC box	−1982(−),−1946(−),−1925(+),−1853(+),−1834(−)	GGCCGCCG/ GGCAGCCG	Ethylene-responsive element
SARE	−1384(+)	TTTTCTTCTC	Salicylic acid-responsive element
GARE	−1804(+)/−573(+)	CCTTTT/ TTTTTTCC	Gibberellin-responsive element
