# Supplementary figures and images for: MePHD1 as a PHD-Finger Protein Negatively Regulates ADP-Glucose Pyrophosphorylase Small Subunit1a Gene in Cassava
Source: Int J Mol Sci. 2018 Sep 19;19(9):2831. doi: 10.3390/ijms19092831 (PMC6164933; doi:10.3390/ijms19092831)

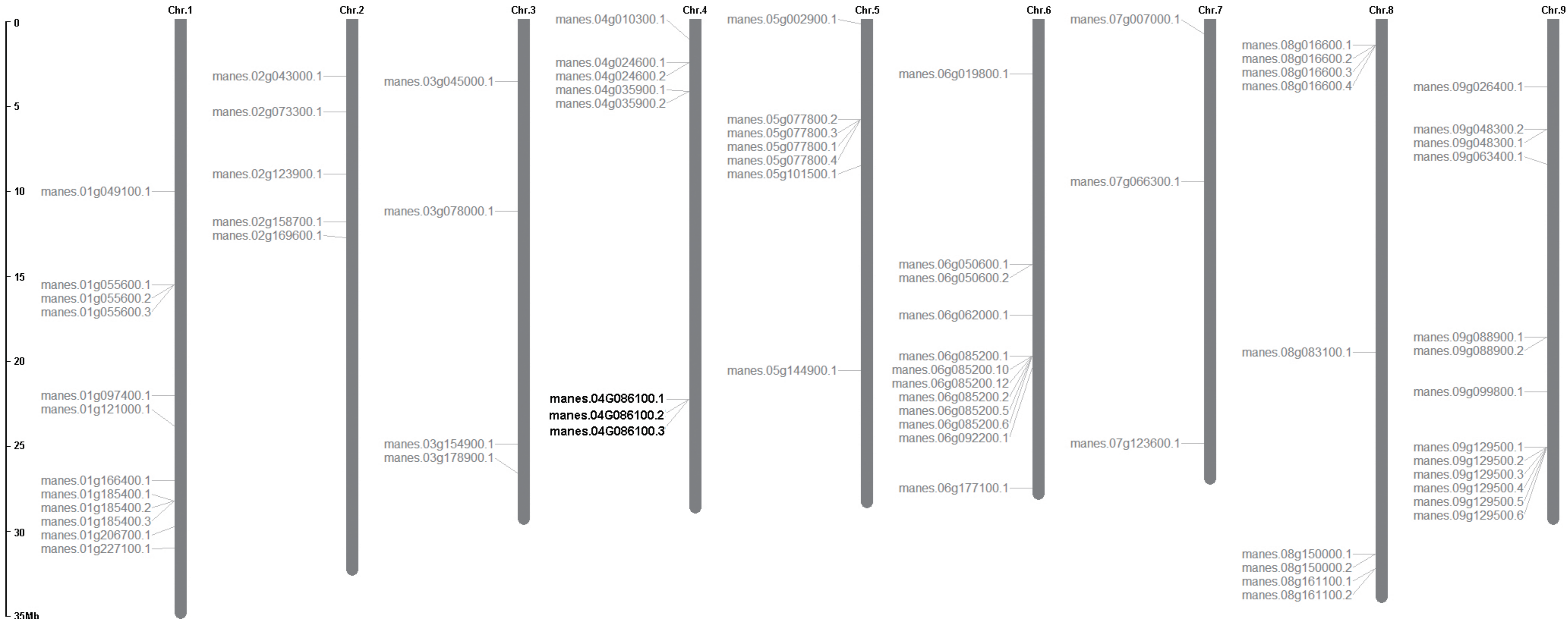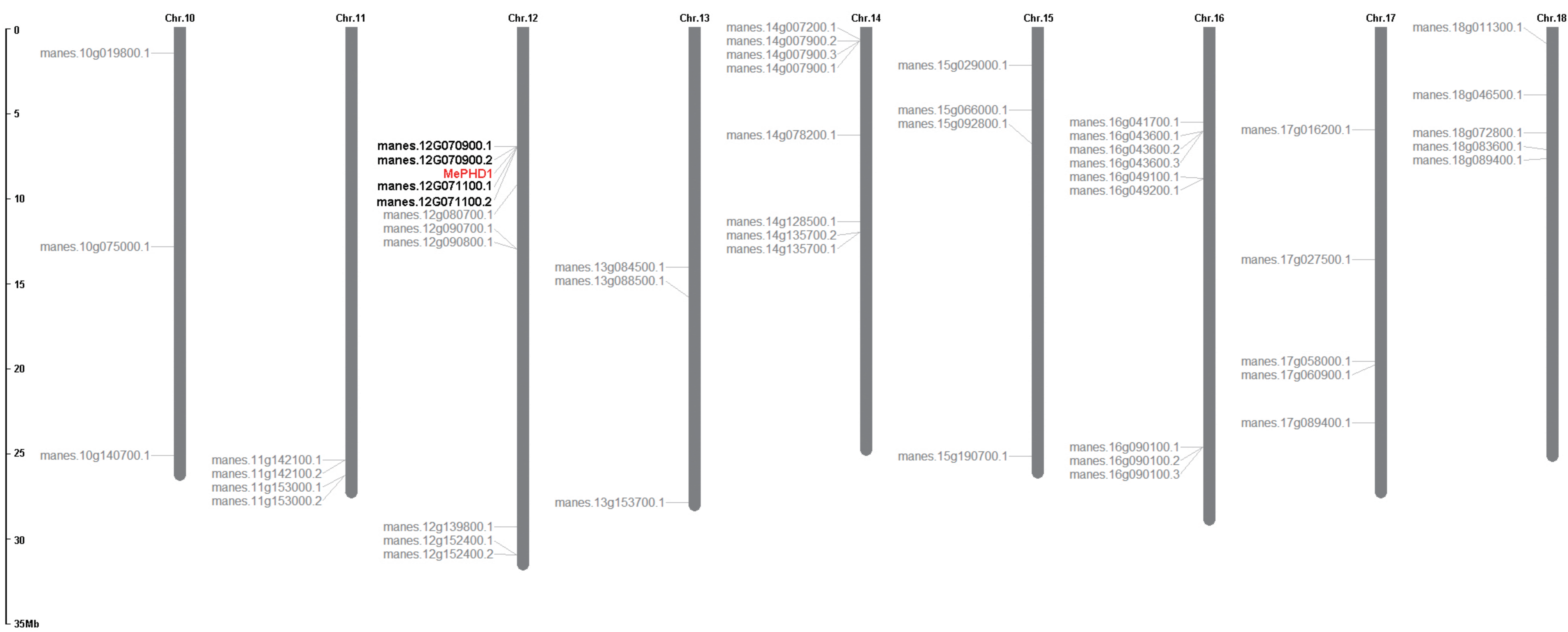

Supplement: Supplementary file 1 [file ijms-19-02831-s001.zip › ijms-345535 Figure S1.pdf]
